# Antitumor activity of HPA3P through RIPK3-dependent regulated necrotic cell death in colon cancer

**DOI:** 10.18632/oncotarget.24083

**Published:** 2018-01-09

**Authors:** Eugene Cho, Jong-Kook Lee, Eunji Park, Chang Ho Seo, Tudor Luchian, Yoonkyung Park

**Affiliations:** ^1^ Department of Biomedical Science, Chosun University, Gwangju, Korea; ^2^ Department of Bioinformatics, Kongju National University, Kongju, Korea; ^3^ Department of Physics, Alexandru I. Cuza University, Iasi, Romania; ^4^ Research Center for Proteinaceous Materials (RCPM), Chosun University, Gwangju, Korea

**Keywords:** HPA3P, peptide, necroptosis, RIPK3, antitumor activity

## Abstract

The antimicrobial peptide HPA3 shows anticancer activity in gastric cancer and leukaemia. However, how HPA3 exerts its anticancer activity, as well as whether it also exhibits activity in other cancers, remains unknown. Therefore, the aim of this study was to evaluate the anticancer activity of HPA3 and its analogues in colon cancer and to elucidate the mechanisms responsible for this activity. HPA3P decreased cell viability, whereas HPA3 and HPA3P2 did not decrease cell viability in colon cancer cells compared with control cells. This reduction in cell viability occurred through necrosis, a conclusion supported by our observation of the release of cellular contents, our intracellular PI staining results, and our observation of the release of HMGB1. Moreover, RIPK3 inhibition blocks the reduction of cell viability by HPA3P. Consistent with this finding, we found that knocking down RIPK3 and MLKL, key necroptosis proteins, attenuates the reductions in cell viability induced by HPA3P. Furthermore, HPA3P can improve the anticancer activity of chemotherapeutic agents and exhibits anticancer activity in other cancer cells. These results suggest that HPA3P may have potential as an anticancer agent in the treatment of colon cancer.

## INTRODUCTION

Cancers can be intrinsically resistant to chemotherapy or may develop resistance to chemotherapy during treatment. Both intrinsic and acquired resistance to anticancer drugs are major causes of treatment failure in patients with cancer. Most anticancer drugs, chemotherapeutic agents (such as 5-FU, gemcitabine, cisplatin, oxaliplatin, and paclitaxel) and molecularly targeted agents (such as imatinib, vemurafenib, and bortezomib) induce apoptosis. However, many cancer cells evade apoptosis by modulating pro- and anti-apoptotic proteins and eventually become resistant to anticancer drugs. Apoptosis evasion by drug resistance is a major factor in cancer cell survival and limits chemotherapy effectiveness [1, 2]. Moreover, despite the performance of increasing numbers of studies regarding pro-apoptotic anticancer drugs, no strategies designed to overcome the limitations of these drugs have been established.

Necrosis is characterized by cytoplasmic swelling and cytoplasmic translucency, as well as the loss of plasma membrane integrity and the release of intracellular contents into the extracellular environment, which usually stimulates inflammation. Necrosis has also been regarded as an uncontrolled form of cell death [3, 4]. However, the results of recent studies indicate that necrosis, like apoptosis, can also occur through a regulated process, namely, necroptosis [5]. Necroptosis is caspase-independent and is mediated by receptor-interacting kinase (RIPK) 1, RIPK3, and mixed lineage kinase domain-like protein (MLKL). RIPK3 is activated to interact with RIPK1 through the RIP homotypic interaction motif, which phosphorylates MLKL as a critical RIPK3 substrate. Activated MLKL subsequently translocates to the plasma membrane, which induces membrane integrity destabilization, leading to cell swelling and membrane rupture [6, 7]. Therefore, necrosis or necroptosis may be a strategy for overcoming resistance of cancer cells to apoptosis.

Antimicrobial peptides (AMPs) are part of the innate immune defence system and exhibit a broad spectrum of antimicrobial effects. They are generally small molecules (12 to 50 amino acids). Fifty percent or more of their composition comprises hydrophobic amino acids. Moreover, AMPs have an overall net positive charge (+2 to +9) resulting from presence of lysine and arginine residues [8, 9]. The membranes of cancer cells and gram-negative and gram-positive bacteria exhibit a negative charge because of the presence of anionic phospholipids, such as phosphatidylserine, lipopolysaccharide, and lipoteichoic acid. However, the membranes of normal mammalian cells exhibit a neutral charge due to the presence of zwitterionic phospholipids [10, 11]. The cationic charge of AMPs is attracted to the anionic components on the surface of the bacterial plasma membrane. This attraction causes bacterial cell membrane permeabilization, which results in plasma membrane disruption characterized by membrane blebbing and vesiculation, as well as the release of intracellular contents. In addition, some peptides bind to intracellular targets by translocating across the bacterial cytoplasmic membrane [12]. Therefore, many AMPs can preferentially disrupt bacterial and cancer cell membranes rather than normal mammalian cell membranes. In addition to reporting on the antimicrobial activity of AMPs, several recent studies have also reported on the anticancer activity of these peptides [13, 14].

The antibacterial peptide HP (2-20) was derived from the amino-terminus of the *Helicobacter pylori* ribosomal protein L1 [15]. This peptide has broad antimicrobial activity against gram-negative bacteria, gram-positive bacteria, and fungi. HPA3, an analogue of HP (2-20), features substitutions of tryptophan for glutamine and aspartic acid at positions 17 and 19, respectively, and consequently exhibits significantly enhanced antimicrobial activity without haemolytic activity [16]. HPA3 has also been modified by the substitution of proline for glutamic acid (HPA3P) at position 9 or by the substitution of proline for glutamic acid and phenylalanine at positions 9 and 12 (HPA3P2), respectively. Consequently, HPA3P displays antimicrobial activity greater than that displayed by HPA3 and HPA3P2 but does not display haemolytic activity. HPA3P is localized in the cytoplasm of bacteria cells and yeast, whereas HPA3 and HPA3P2 are localized on the bacterial membrane surface [17, 18]. HPA3 has anticancer activity against gastric cancer and acute myelogenous leukaemia [16], but the anticancer activity of HPA3P and HPA3P2 has not been reported. Therefore, in the present study, the anticancer activity of these peptides against colon cancer cells was assessed, and the mechanisms underlying the anticancer activity of the peptides were also investigated.

## RESULTS

### HPA3P-induced human colon cancer cell death is not apoptosis

**Figure 1:**
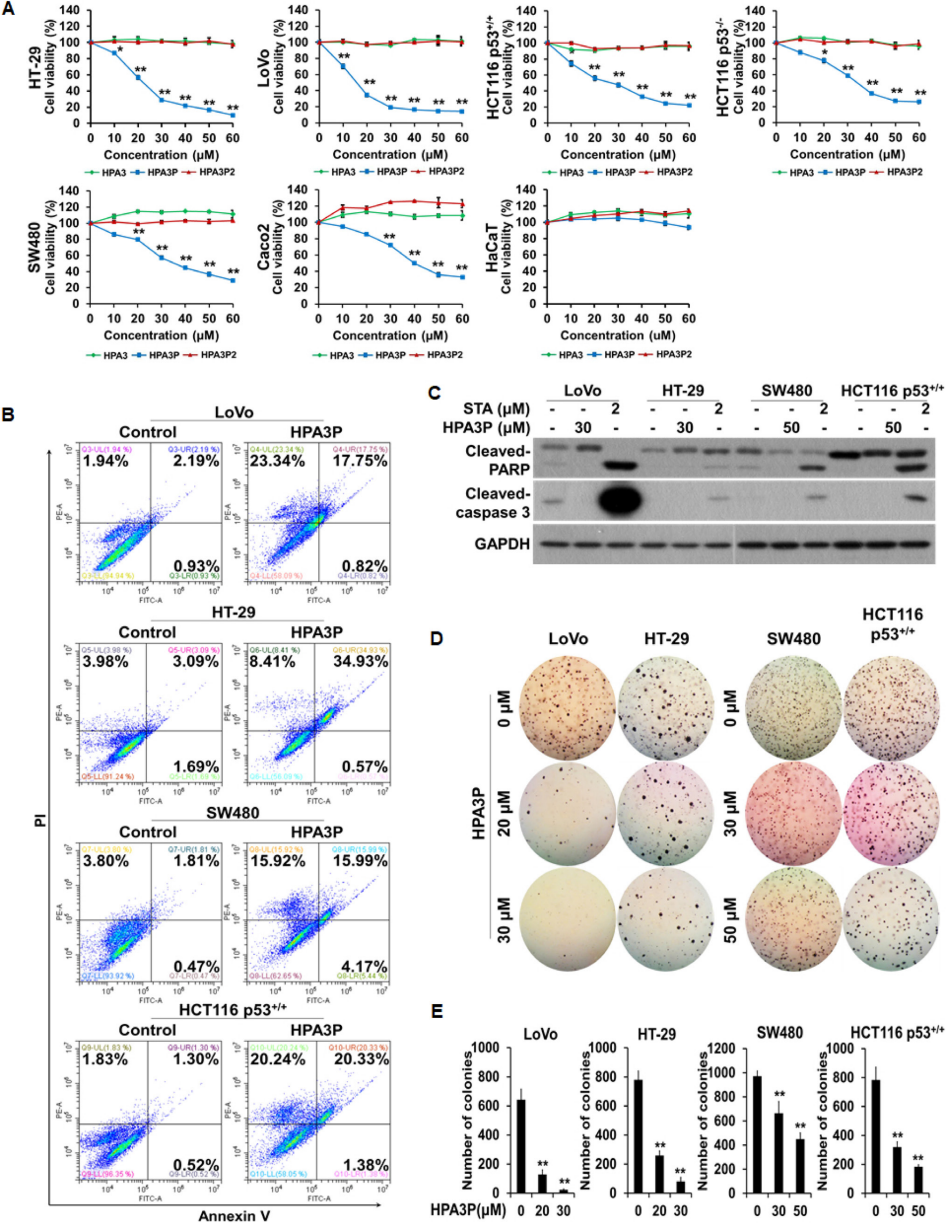
HPA3P induces cell death in human colon cancer cells (**A**) All of the colon cancer cell lines were treated with different concentrations of HPA3, HPA3P, and HPA3P2 for 24 h. The effects of HPA3, HPA3P, and HPA3P2 on cell viability in the indicated colon cancer cell lines were measured by MTT assay. The data are shown as the mean ± SEM. ^*^p < 0.05 and ^**^p < 0.01 compared with control. (**B**) Cell death induction in colon cancer cell lines treated with HPA3P (LoVo and HT-29, 30 μM; SW480 and HCT116 p53^+/+^, 50 μM) was assessed by flow cytometry using annexin V and PI. (**C**) All cells were treated with the indicated concentrations of HPA3P for 24 h. All cell lines were treated with staurosporine, which served as a positive control. Whole-cell lysates were prepared, and apoptosis was assessed by western blot analysis using anti-cleaved caspase-3, anti-cleaved PARP, and GAPDH antibodies. (**D**) Anchorage-independent growth in the HPA3P-treated colon cancer lines was assessed by colony formation assay. Colony formation was observed 10 days after plating. Images were photographed using a camera attached to a Nikon SMZ800 stereomicroscope (magnification, 4×). (**E**) Statistical analysis was performed to quantify relative colony formation in the HPA3P-treated and non-treated cell lines. Colonies were counted using ImageJ software. The results represent the average number of colonies from three replicated experiments. The data are shown as the mean ± SD. ^**^p < 0.01 compared with control.

To investigate the effects of HPA3, HPA3P, and HPA3P2 on cell viability in colon cancer cell lines, we performed an MTT assay. We found that cell viability decreased significantly with increasing HPA3P concentrations in six colon cancer cell lines. However, no decrease in cell viability was observed in the normal cell line, i.e., the HaCaT cell line, when these cells were treated with HPA3P. HPA3 and HPA3P2 had no effects on cell viability in these cell lines (Figure 1A). To determine whether the abovementioned HPA3P-induced reductions in cell viability in the LoVo, HT-29, SW480, and HCT116 p53^+/+^ cell lines were related to apoptotic cell death, we performed flow cytometry analysis. The numbers of annexin V-positive/PI-positive and PI-positive cells were significantly increased in the HPA3P-treated cell line compared with the non-treated cell line. However, no annexin V-positive and PI-negative cells were detected in the HPA3P-treated cell lines (Figure 1B). Caspase 3 is activated by caspase 9, and PARP is cleaved by activated caspase 3. These are well-characterized apoptotic events [19]. Therefore, to determine whether HPA3P can induce apoptosis in colon cancer cell lines, we assessed cleaved-caspase 3 and PARP expression by western blotting. Cleaved-caspase 3 and cleaved-PARP were not detected in HPA3P-treated cells but were detected in staurosporine-treated cells (Figure 1C and Supplementary Figure 4A). Staurosporine is a well-known apoptosis inducer in a wide range of cells. Since cancer cell colony formation is closely related to cancer cell growth, we investigated the effects of HPA3P on colon cancer cell anchorage-independent growth by colony formation assay. We found that colon cancer cell colony formation ability was significantly reduced by HPA3P (Figure 1D and 1E). These results indicate that HPA3P-mediated reductions in cell viability and cell growth inhibition are caused by a type of cell death other than apoptosis.

### HPA3P-induced human colon cancer cell death is necrosis

**Figure 2:**
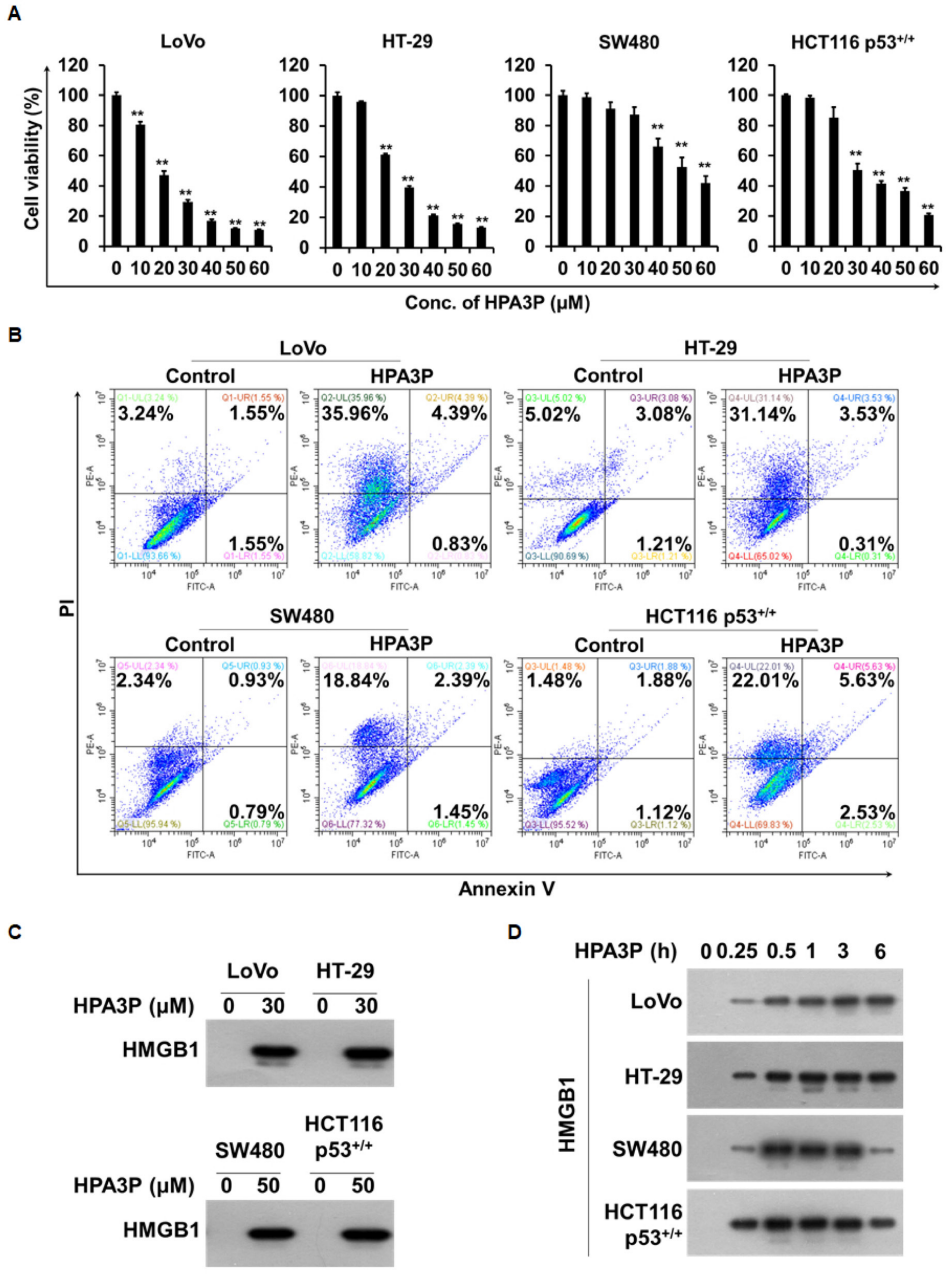
HPA3P-induced human colon cancer cell death is necrosis (**A**) The indicated cell lines were treated with different concentrations of HPA3P for 6 h. The effects of HPA3P on cell viability in the colon cancer cell lines were measured by MTT assay. The data are shown as the mean ± SEM. ^**^p < 0.01 compared with control. (**B**) The indicated cell lines were treated with HPA3P (LoVo and HT-29, 30 μM; SW480 and HCT116 p53^+/+^, 50 μM) for 6 h. Necrotic cell death was assessed by flow cytometry using annexin V and PI. (**C**) The cells were treated with the indicated concentration of HPA3P for 6 h. (**D**) The indicated cell lines were treated with HPA3P (LoVo and HT-29, 30 μM; SW480 and HCT116 p53^+/+^, 50 μM) and then incubated for the indicated time. Culture media were precipitated with TCA. The samples were assessed by western blot analysis using an anti-HMGB1 antibody. Six hours was used as a positive control time.

To investigate the effects of HPA3P-induced rapid cell death on cell viability in the above colon cancer cell lines, we performed MTT assay. We found that cell viability was significantly reduced in a concentration-dependent manner in the colon cancer cell lines within 6 h of HPA3P administration (Figure 2A). To determine whether this HPA3P-induced rapid cell death was necrotic cell death, we performed flow cytometry analysis. There were no differences in the numbers of annexin V-positive/PI-positive and annexin V-positive/PI-negative cells between the HPA3P-treated cell lines and the non-treated cell lines after 6 h of treatment with HPA3P. However, the percentage of PI-positive cells in the HPA3P-treated cell lines was significantly higher than that in the non-treated cell lines (Figure 2B). To determine whether HPA3P was able to induce the release of high mobility group protein B1 (HMGB1), an indicator of necrosis, in colon cancer cell lines, we performed western blot analysis in cell culture media. The results of this analysis showed that HMGB1 was passively released from the indicated colon cancer cell lines by HPA3P (Figure 2C and Supplementary Figure 4B). In addition, HMGB1 release was observed at an early time in cell culture media (Figure 2D and Supplementary Figure 4C). Taken together, our PI staining and HMGB1 release results indicate that HPA3P disrupts the cell membrane, suggesting that the cell death induced by HPA3P is necrosis.

### HPA3P induces rapid cell necrosis

**Figure 3:**
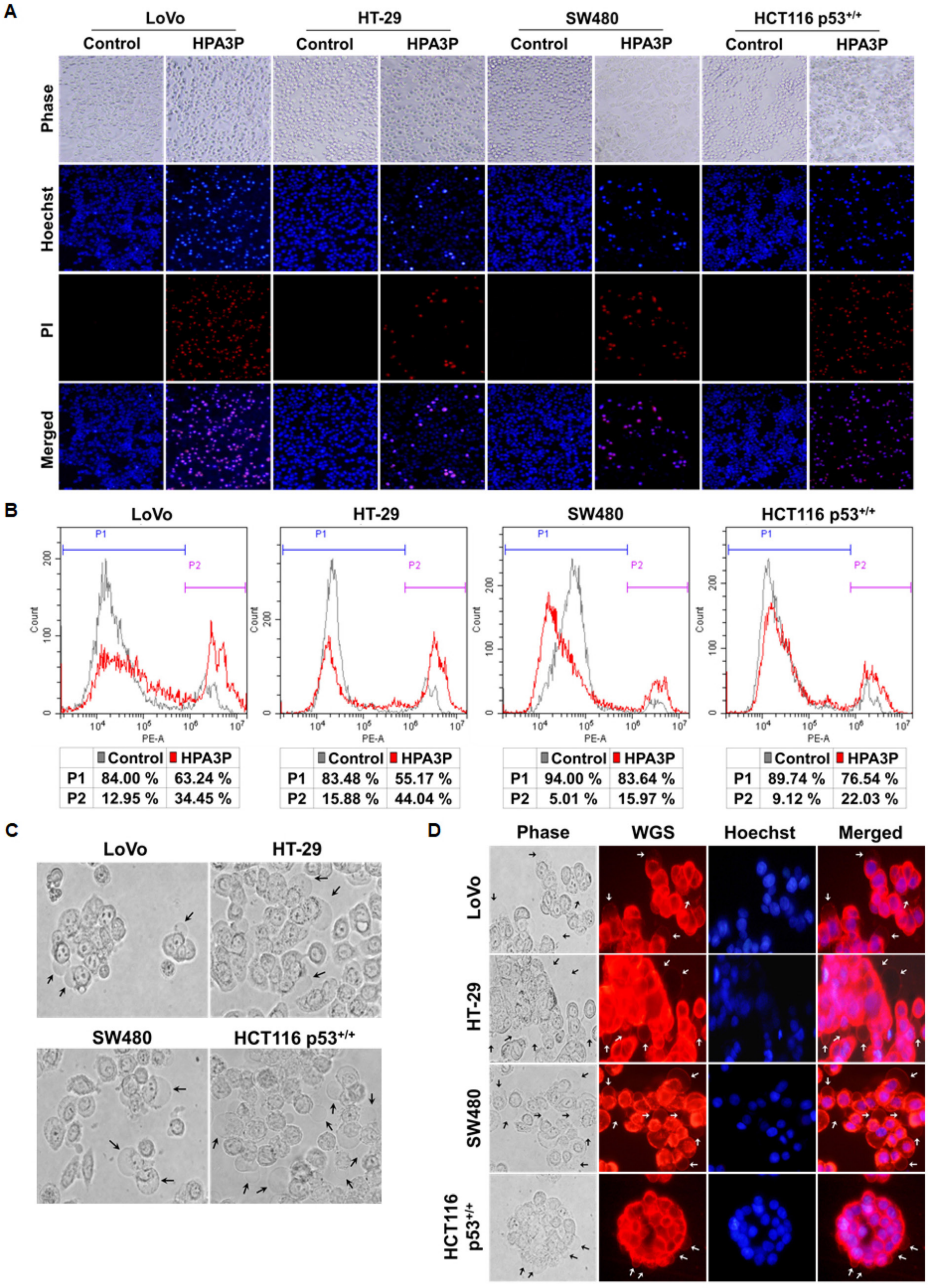
HPA3P-induced necrosis and morphological changes in human colon cancer cell lines All cell lines were treated with HPA3P for 30 min and then stained with Hoechst 33342/PI or PI alone. (**A**) Pictures of Hoechst 33342/PI-stained cells were taken using an Olympus IX71 fluorescence microscope (magnification LCAch N 20X). (**B**) PI-stained cells were analysed by flow cytometry. (**C**) Morphological changes in colon cancer cells treated with HPA3P for 5 min were observed using an Olympus IX71 light microscope (magnification LUCPlaFL N 40X). The black arrows indicate membrane blebs. (**D**) The cells were incubated with wheat germ agglutinin (WGS) and Hoechst 33342 for 10 min before being washed and then treated HPA3P for 5 min. Morphological changes were observed under an Olympus IX71 fluorescence microscope (magnification LUCPlaFL N 40X). The cell membrane and nucleus were specifically stained by wheat germ agglutinin conjugated with Alexa Fluor 594 (red) and Hoechst 33342 (blue), respectively. The black and white arrows indicate membrane blebs. The LoVo and HT-29 cell lines were treated with 30 μM, and the SW480 and HCT116 p53^+/+^ cell lines were treated with 50 μM HPA3P.

To investigate the phenomenon of HPA3P-mediated cell membrane permeabilization in colon cancer cell lines, we performed PI staining in live cell lines. Cell membrane permeabilization was observed mostly in the HPA3P-treated cell lines over 30 min (Figure 3A). In addition, the numbers of PI-stained cells were increased in the HPA3P-treated cell lines compared with the non-treated cell lines (Figure 3B). To investigate the colon cancer cell morphological changes induced by HPA3P, we observed the HPA3P-treated cell lines for 5 min via fluorescence microscopy. We observed cell membrane blebbing in the LoVo, HT-29, SW480, and HCT116 p53^+/+^ cell lines and in the WGA-stained cell lines (Figure 3C and 3D). Additionally, cytoplasmic swelling, cell membrane rupture, and intracellular contents release in these cell lines (Supplementary Video 1). These results suggest that HPA3P-induced necrosis occurs rapidly through membrane disruption.

### HPA3P has penetration ability and the effects of the combination of HPA3P and anticancer drugs

**Figure 4:**
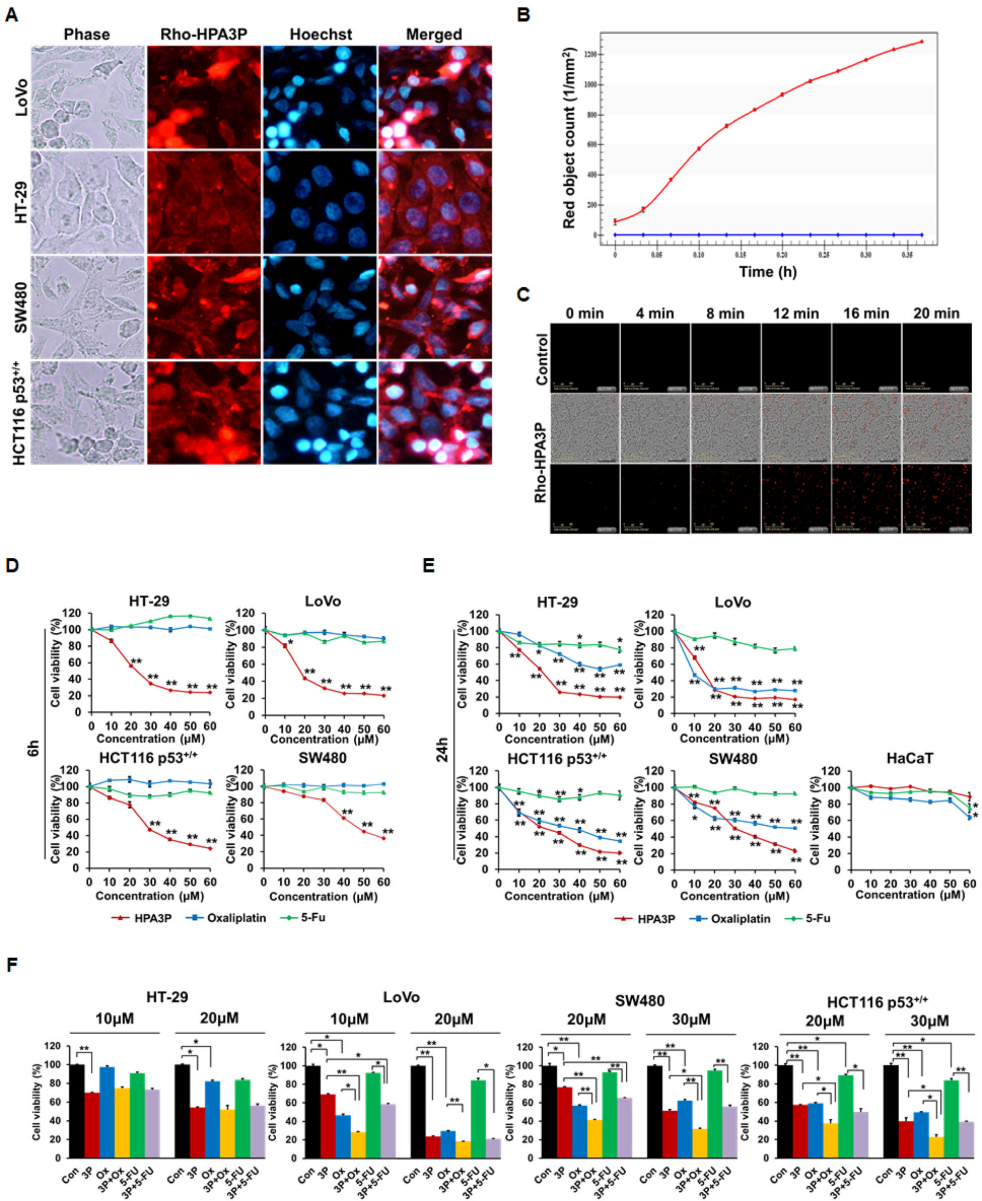
Intracellular localization of HPA3P and the effects of the combination of HPA3P and anticancer drugs (**A**) All cell lines were treated with rhodamine-labelled HPA3P (LoVo and HT-29, 30 μM; SW480 and HCT116 p53^+/+^, 50 μM) for 5 min. The cells were washed and then stained with Hoechst 33342 before being observed under an Olympus IX71 fluorescence microscope (magnification PlanC N 40X). (**B**, **C**) HCT116 p53^+/+^ cells were treated with 30 μM of rhodamine-labelled HPA3P. Fluorescence was measured in four different areas per well for approximately 20 minutes using an IncuCyte ZOOM. The CI values are presented in Supplementary Table 1. The cells were treated with different concentrations of HPA3P, oxaliplatin and 5-FU for 6 h (**D**) or 24 h (**E**). (**F**) The cells were treated with the indicated concentrations of HPA3P (3P), oxaliplatin (Ox), and 5-Fu for 24 h. Cell viability was measured by MTT assay. Data are shown as the mean ± SEM. ^*^p < 0.05 and ^**^p < 0.01.

To gain insight into the mechanism underlying the anticancer effects of HPA3P in colon cancer cell lines, we evaluated the distribution of HPA3P in colon cancer cell lines via fluorescence microscopy. HPA3P was rapidly and easily internalized by colon cancer cell lines within 5 min of its administration and was distributed in the cytoplasm. However, it was not possible to clearly determine whether HPA3P is located only in the cytoplasm or nucleus in the HT-29 cell line (Figure 4A). Therefore, to determine whether HPA3P is located exclusively in the cytoplasm or nucleus in the LoVo and HT-29 cell lines, we compared its localization patterns in the two cell lines using confocal microscopy. The results of this examination showed that HPA3P was localized only in the cytoplasm in LoVo and HT-29 cells (Supplementary Figure 1). Then, we analysed the ability of HPA3P to penetrate cells using an IncuCyte ZOOM real-time live-cell imaging system. The numbers of living cells stained with rhodamine-labelled HPA3P increased significantly within minutes of HPA3P administration in the HCT116 p53^+/+^ cell population (Figure 4B, 4C and Supplementary Video 2). To compare the effects of HPA3P and anticancer drugs (e.g., oxaliplatin and 5-FU) on cell viability in the above colon cancer cell lines, we performed MTT assay. The results of this assay showed that cell viability decreased significantly in HPA3P-treated cells over 6 h; however, oxaliplatin and 5-FU had no effect on cell viability (Figure 4D). In addition, HPA3P induced a marked decrease in cell viability in treated cells compared with non-treated cells over 24 h. Oxaliplatin induced a significant decrease in cell viability in LoVo, SW480, and HCT116 p53^+/+^ cells but reduced cell viability only slightly in HT-29 cells over 24 h. 5-FU had no effect on cell viability in these cell lines over 24 h (Figure 4E). To investigate the combined effects of HPA3P, oxaliplatin, and 5-FU on cell viability, we subsequently performed MTT assay after the indicated combination treatment. The results of the assay showed that cell viability was significantly reduced in the LoVo, SW480, and HCT116 p53^+/+^ cell lines treated with the combination of HPA3P and oxaliplatin compared with those treated HPA3P or oxaliplatin alone. However, the combination of HPA3P and 5-FU decreased cell viability only slightly in the LoVo and SW480 cell lines. Cell viability was not different between HT-29 cells treated with the combination of HPA3P and oxaliplatin or 5-FU, indicating that HT-29 cell viability was reduced only by HPA3P (Figure 4F). Taken together, these data suggest that HPA3P exhibits activity after rapidly penetrating the cell and entering the cytoplasm and exhibits improved anticancer activity at a lower concentration when combined with oxaliplatin.

### HPA3P can trigger RIPK3-dependent necroptosis

**Figure 5:**
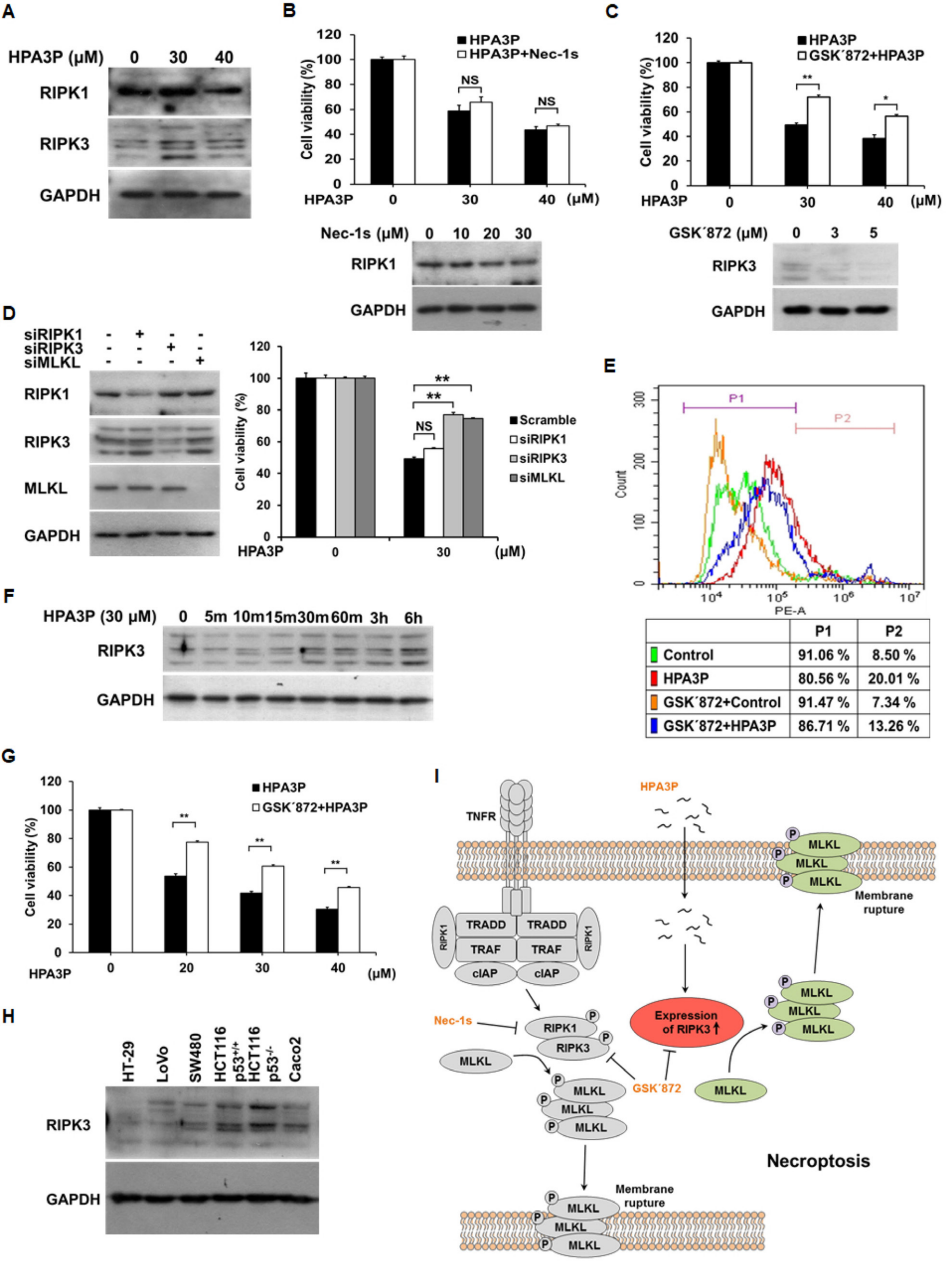
Low concentrations of HPA3P induce RIPK3-dependent necroptosis in HCT116 p53^+/+^ cells (**A**) HCT116 p53^+/+^ cells were treated with HPA3P for 6 h. The cell lysates were analysed for RIPK1 and RIPK3 expression by western blotting. The cells were pretreated with 30 μM Nec-1s (**B**) or 3 μM GSK΄872 (**C**) for 2 h and (**D**) transfected with negative-control (scramble), RIPK1, RIPK3, and MLKL siRNA for 24 h. The cells were subsequently treated with HPA3P. HPA3P-induced cytotoxicity was measured by MTT assay. (B–D) The cells were treated with Nec-1s or GSK΄872, and transfected with siRNA for 24 h. The cell lysates were analysed by western blot analysis. (**E**) The cells were treated with 3 μM GSK΄872 for 2 h and subsequently treated with 30 μM HPA3P for 6 h. The cells were stained with PI for flow cytometric analysis. (**F**) The cells were treated with 30 μM HPA3P for the indicated times. RIPK3 expression was analysed by western blotting. (**G**) The cells were pretreated with 3 μM GSK΄872 for 2 h and then treated with HPA3P for 24 h. Cell viability was measured by MTT assay. ^*^p < 0.05, ^**^p < 0.01 indicate a significant difference between the indicated groups. NS, not significant. (**H**) The colon cancer cell lysates used in this study were analysed by western blotting. (**I**) Schematic diagram of HPA3P-induced necroptosis.

Necroptosis shares many features with necrosis, including membrane rupture and cytoplasmic swelling, as well as the release of intracellular contents [20, 21]. Thus, to determine whether HPA3P can induce necroptosis, we measured the expression of RIPK1 and RIPK3, key necroptosis proteins, in HPA3P-treated HCT116 p53^+/+^ cells. RIPK3 expression increased significantly in HPA3P-treated cells, while RIPK1 expression seemed to increase slightly in 30 μM HPA3P-treated cells (Figure 5A and Supplementary Figure 4F). HPA3P-induced increases in RIPK3 expression were inhibited by GSK΄872 (an RIPK3 inhibitor) and RIPK3 siRNA, resulting in restored cell viability. However, Nec-1s (an RIPK1 inhibitor) and RIPK1 siRNA did not restore cell viability. We also observed that MLKL siRNA-treated cells displayed restored cell viability compared with negative-control siRNA-treated cells (Figure 5B–5D and Supplementary Figure 4D, 4E and 4G). In addition, the number of PI-stained cells was decreased in cells treated with GSK΄872 compared with those treated with HPA3P alone (Figure 5E). HPA3P rapidly penetrates cells, enters the cytosol within minutes of its administration (Figure 4B) and rapidly induces necrosis (Figure 2D and Figure 3D). Therefore, to confirm that HPA3P rapidly induces RIPK3 expression, we performed western blot analysis. The results of this analysis showed that RIPK3 expression began to increase at 10 minutes after HPA3P treatment and increased significantly within 30 minutes after HPA3P treatment (Figure 5F and Supplementary Figure 4H). Furthermore, as cell viability was also reduced at 24 hours after 20 μM HPA3P treatment, we investigated the protective effects of GSK΄872 on HPA3P-mediated cytotoxicity using MTT assay. Similar to the results shown in Figure 5C, the results of this experiment showed that GSK΄872 inhibited HPA3P-induced cytotoxicity (Figure 5G). Finally, we measured RIPK3 expression in the colon cancer cells used in this experiment and confirmed that protein and mRNA of RIPK3 were expressed in all cells (Figure 5H and Supplementary Figure 4I). These results suggest that low concentrations of HPA3P lead to RIPK3-dependent necroptosis.

### HPA3P inhibits xenograft tumour growth *in vivo*

**Figure 6:**
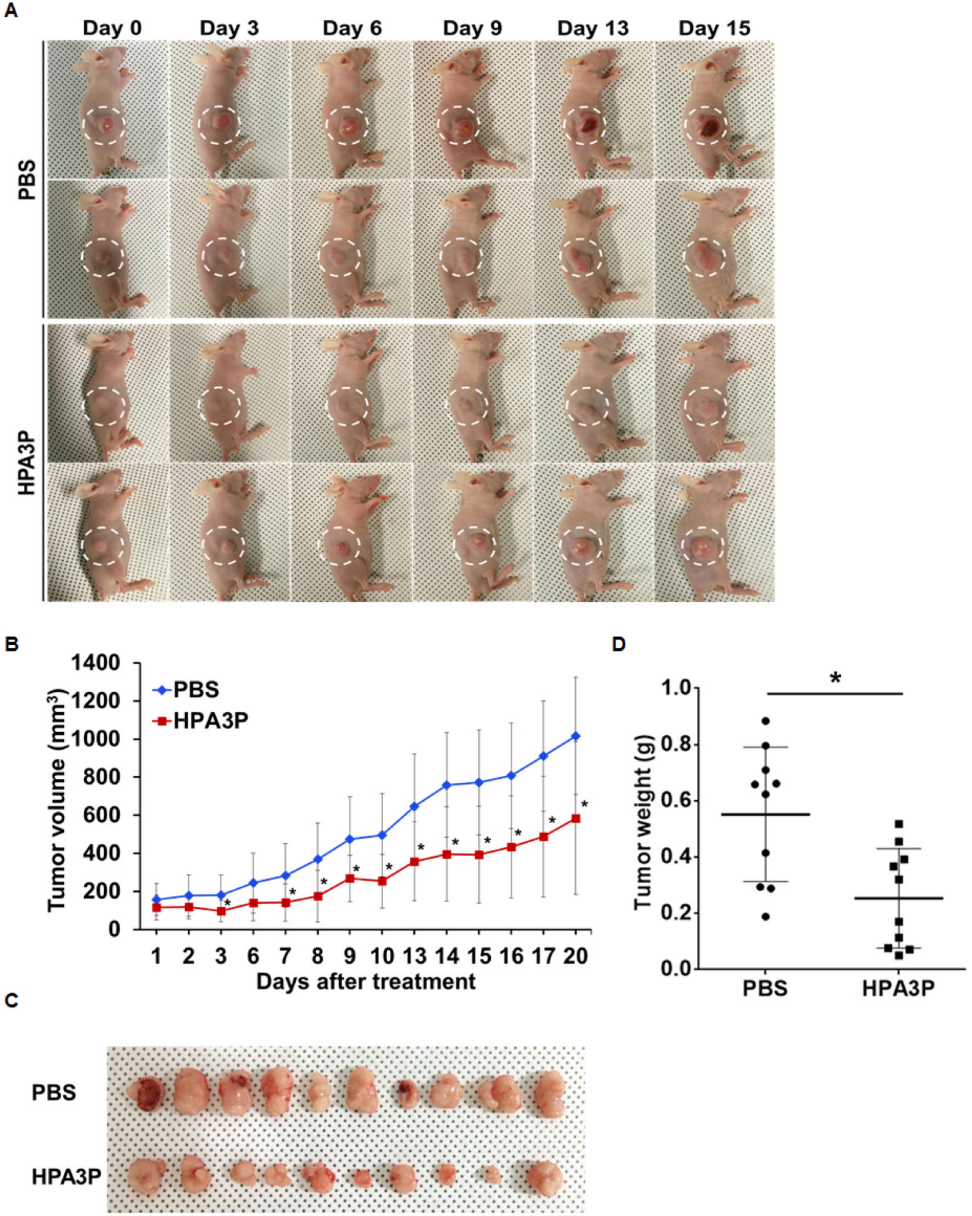
HPA3P inhibits tumour growth in mouse tumour models HCT116 p53^+/+^ cells were injected into the right flanks of the nude mice. Ten days after implantation, the mice were treated with 25 mg/kg HPA3P and PBS by intravenous injection. (**A**) Representative images were photographed, and (**B**) tumour volumes were measured on the indicated date. The data are presented as the mean ± SD, *n* = 10 per group. ^*^p < 0.05. (**C**) Images were obtained, and (**D**) tumour weights were measured on day 20 after tumour resection. The tumour weights are presented as scatter dot data. The data are presented as the mean ± SD, *n* = 10 per group. ^*^p < 0.05 compared with PBS.

To validate the antitumour effects of HPA3P on *in vivo* tumour growth using mouse xenograft models, we administered 25 mg/kg HPA3P via intravenous injection for 20 days. The results of this experiment showed that HPA3P inhibited tumour growth (Figure 6A). On day 20, the tumour volume and tumour weight had decreased by approximately 42% and 54% compared with control, respectively (Figure 6B–6D). In addition, the expression of RIPK3 was increased in tumour tissue extract of HPA3P-treated mice, which is consistent with an increase in RIPK3 by HPA3P in colon cancer cells (Supplementary Figure 3 and Supplementary Figure 4K). These results suggest that HPA3P probably has potential as a therapeutic agent.

### HPA3P induces necrosis in other cancer cell lines

**Figure 7:**
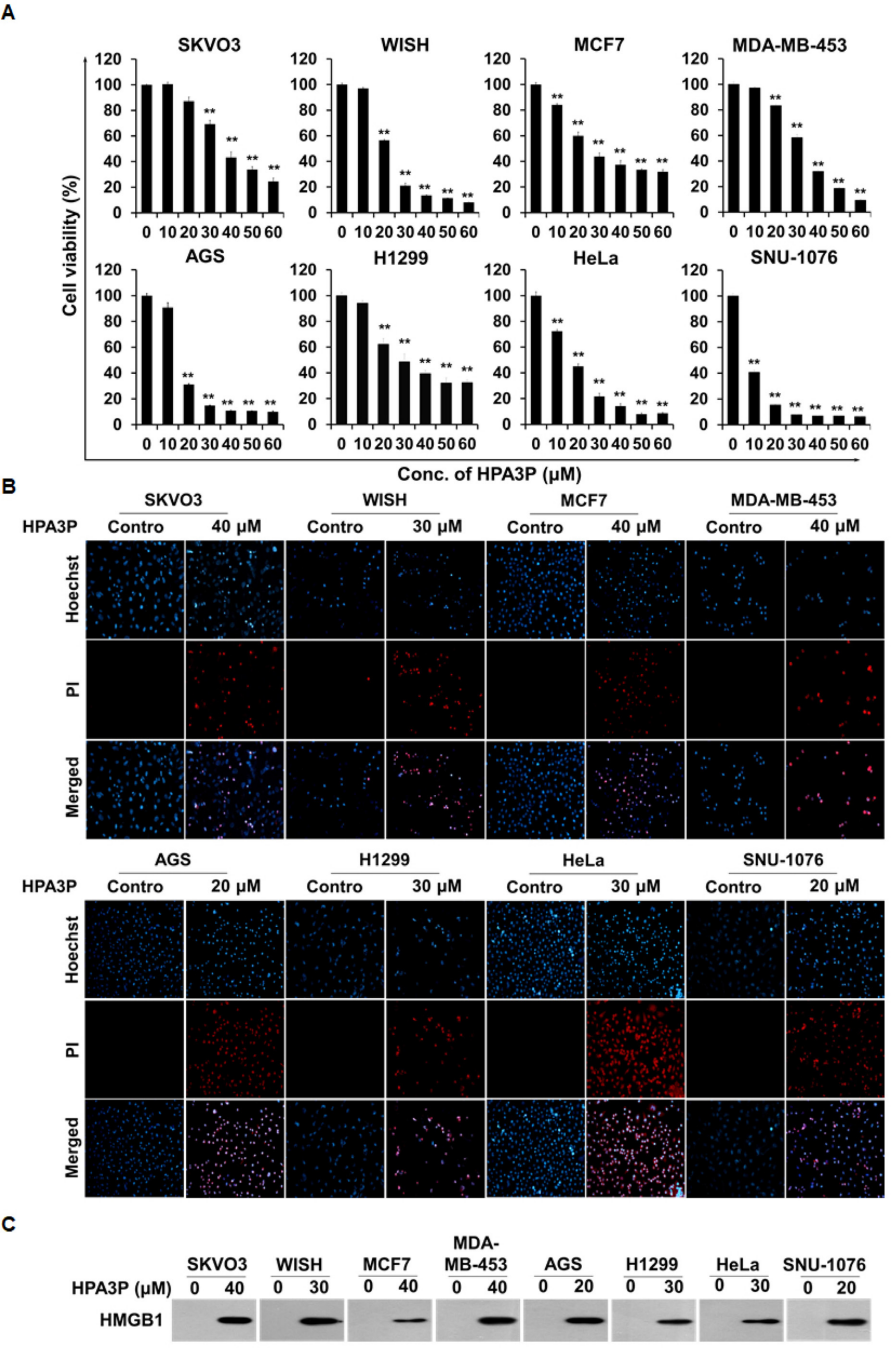
HPA3P induces necrosis in other cancer cell lines (**A**) SKVO3, WISH, MCF7, MDA-MB-453, AGS, H1299, HeLa, and SNU-1076 cells were treated with various concentrations of HPA3P for 6 h. HPA3P-induced cytotoxicity was assessed by MTT assay. The data are shown as the mean ± SEM. ^**^p < 0.01 compared with control. (**B**) SKVO3, WISH, MCF7, MDA-MB-453, AGS, H1299, HeLa, and SNU-1076 cells were treated with the indicated concentration of HPA3P for 30 min. All cell lines were stained with PI and Hoechst 33342. Images were taken using an Olympus IX71 fluorescence microscope (magnification LCAch N 20X). (**C**) SKVO3, WISH, MCF7, MDA-MB-453, AGS, H1299, HeLa, and SNU-1076 cells were treated with the indicated concentration of HPA3P for 6 h. Culture media were precipitated with TCA. The samples were assessed by western blot analysis using an anti-HMGB1 antibody.

To investigate the effects of HPA3P on cell viability in other cancer cell lines, we performed MTT assay. The results of this assay showed that cell viability decreased significantly in human ovarian carcinoma (SKOV3), human HeLa cell-contaminated amnion (WISH), human breast adenocarcinoma (MCF7), human breast metastatic carcinoma (MDA-MB-453), human gastric adenocarcinoma (AGS), human non-small lung carcinoma (H1299), human cervical carcinoma (HeLa), and human laryngeal squamous cell carcinoma (SNU-1076) cells within 6 h of HPA3P administration (Figure 7A). Moreover, cell membrane permeabilization occurred in HPA3P-treated cell lines, as demonstrated by PI staining, but did not occur in non-treated cell lines (Figure 7B). To assess HMGB1 release in these cell lines, we subsequently performed western blot analysis in cell culture media. We measured HPA3P-induced HMGB1 release into culture media in the indicated cell lines (Figure 7C and Supplementary Figure 4J). The results of this experiment suggest that HPA3P also has anticancer activity in other cancer cell lines.

## DISCUSSION

A growing number of studies suggest that cationic AMPs can kill cancer cells without having cytotoxic effects on normal cells, indicating that AMPs may have potential as novel anticancer agents [13]. HPA3 was derived from HP (2-20), which was modified by the substitution of proline for glutamic acid at position 9 (HPA3P) or by the substitutions of proline for glutamic acid and phenylalanine at position 9 and 12 (HPA3P2), respectively. These peptides exhibit antimicrobial activity without haemolytic activity but exert their effects by different mechanisms of action, such as pore formation, membrane permeation and membrane component modification [18]. The anticancer activity of these peptides has not been reported in colorectal cancer; however, the anticancer activity of HPA3 has been reported in gastric cancer and acute myelogenous leukaemia [16]. HPA3P induced reductions in cell viability in the abovementioned colon cancer cell lines, but HPA3 and HPA3P2 did not affect cell viability in those cell lines. None of the peptides had cytotoxic effects on normal human keratinocytes. Moreover, HPA3P did not induce haemolysis at concentrations up to 800 μM in human red blood cells [18]. Annexin V positive/PI positive and PI positive cells were significantly increased, but cleaved-caspase 3 and PARP were not detected in HPA3P-treated cell lines. Taken together, these findings indicate that HPA3P-induced cell death probably occurs as a result of necrosis rather than apoptosis. Anchorage-independent growth was also eventually reduced in colon cancer cell lines.

Cationic AMPs have been shown to damage the cell membrane within minutes of their administration [22–24]. We observed a rapid decline in cell viability, an increase in the number of PI-stained cells, and intracellular HMGB1 release soon after HPA3P administration. HMGB1 was passively released from necrotic cells [25]. In addition, HPA3P induced increases in PI uptake, cell membrane blebbing, and cytoplasmic swelling, as well as the spilling of intracellular contents, by disrupting the cell membrane (Supplementary Video 1). These findings support the hypothesis that HPA3P-induced cell death is necrotic cell death. Moreover, HPA3P-induced rapid PI uptake, reductions in cell viability, and HMGB-1 release have also been observed in human ovarian, breast, gastric, non-small lung, cervical, laryngeal squamous cancer, and HeLa cell-contaminated amnion cells (Figure 7).

HPA3P accumulates in the cytoplasm and inhibits protein synthesis by binding to DNA after penetrating the bacterial cell membrane [18]. Generally, bacterial membranes exhibit a negative charge as a result of the presence of anionic phospholipids on their surface, and cationic AMPs are selectively attracted to these anionic phospholipids. Cancer cell membranes also possess negative charges as a result of the presence of several anionic molecules, such as phosphatidylserine, sialic acid, membrane-associated glycoproteins. In contrast, normal cell membranes usually exhibit a neutral charge due to the presence of zwitterionic phospholipids [10, 26]. Thus, we surmised that HPA3P may penetrate cancer cells without affecting normal cells. Our results show that HPA3P rapidly penetrates colon cancer cells and accumulates in the cytoplasm (Figure 4 and Supplementary Figure 1).

5-FU is generally administered as an adjuvant in systematic colorectal cancer treatment [27]. Oxaliplatin causes impaired DNA replication and apoptosis [28]. In addition, oxaliplatin and 5-FU have synergistic effects in colorectal cancer patients [29, 30]. However, unlike other chemotherapeutic agents used for the treatment of colorectal cancer, oxaliplatin induces acute and chronic neurosensory symptoms and paresthesias [31]. In addition, most anticancer drugs induce apoptosis, and many cancer cells evade apoptosis and eventually acquire resistance to apoptosis-inducing anticancer drugs [1, 2]. Therefore, drugs that can overcome resistance to anticancer drug-induced apoptosis are necessary. Combination therapy is one way of overcoming resistance to cancer drug-induced apoptosis. The combination of low concentration of HPA3P and oxaliplatin reduced cancer cell viability more effectively than either agent alone, but the combination of low concentration of HPA3P and 5-FU did not effectively reduce cell viability. These results indicate that the combination of HPA3P and oxaliplatin can reduce oxaliplatin-induced cytotoxicity and may be able to overcome the limitations of apoptosis-inducing anticancer drugs.

HPA3P has rapid anticancer effects; however, these effects cannot be controlled. The morphological features of necroptosis are similar to those of necrosis, but necroptosis is regulated differently than necrosis. Therefore, the ability of HPA3P to induce necroptosis may enable the compound to overcome the limitations of necrosis. Our results show that RIPK1 and RIPK3 expression levels were slightly and significantly increased by low concentrations of HPA3P, respectively. However, HPA3P-induced reductions in cell viability were not attenuated by the RIPK1 inhibitor. Interestingly, when RIPK3 and MLKL expression levels were reduced, the decreases in cell viability induced by HPA3P were restored. Furthermore, RIPK3 expression levels responded rapidly to HPA3P. These findings support the idea that HPA3P can induce RIPK3-dependent necroptosis rather than RIPK1-dependent necroptosis and can thus regulate cell death. Our findings regarding RIPK3 expression in HCT116 p53^+/+^ cells were not consistent with those of previous reports, which showed that RIPK3 is not expressed in the indicated cell line [32, 33]. In contrast, in studies using columbianadin and 11′-deoxyverticillin A, RIPK3 was reported to be expressed in HCT116 cells [34, 35]. Our results show that protein and mRNA of RIPK3 were expressed in HCT116 p53^+/+^ cells and in other colon cancer cells, findings supported by our experiments involving specific siRNAs and inhibitors (Figure 5 and Supplementary Figure 2).

Taken together, our findings indicate that HPA3P induces antitumour effects in mouse models, colon cancer cells, and other cancer cells. Therefore, HPA3P has potential as a therapeutic agent, as it can overcome the limitations of apoptosis-inducing anticancer drugs by inducing necroptosis and can exert enhanced anticancer effects when used in combination with other anticancer drugs.

## MATERIALS AND METHODS

### Cell culture

HT-29 and LoVo (human colorectal adenocarcinoma), AGS (human gastric adenocarcinoma), HeLa (human cervical carcinoma), WISH (human HeLa cell-contaminated amnion), and SNU-1076 (human laryngeal squamous cell carcinoma) cells were cultured in RPMI-1640 medium. SW480, Caco2 (human colorectal adenocarcinoma), HCT116 p53^+/+^, HCT116 p53^−/−^ (human colorectal carcinoma), MCF7 (human breast adenocarcinoma), MDA-MB-453 (human breast metastatic carcinoma), H1299 (human non-small lung carcinoma), SKOV3 (human ovarian carcinoma) and HaCaT (human immortal keratinocytes) cells were cultured in DMEM. The cells were maintained at 37°C in a humidified atmosphere of 5% CO_2_. All media were supplemented with 10% heat-inactivated foetal bovine serum, 100 U/ml penicillin and 100 μg/ml streptomycin (WELGENE, South Korea). The Caco2, HCT116 p53^+/+^, and HCT116 p53^−/−^ cell lines were supplied by Dr. Jong-Suk Kim (Chonbuk National University).

### Reagents

HPA3, HPA3P, and HPA3P2 were synthesized as described previously [18]. Oxaliplatin (O9512) and 5-FU (F6627) were purchased from Sigma Aldrich (MO, USA). The peptides and oxaliplatin were dissolved in PBS. 5-FU was dissolved in DMSO, and then aliquots of the reagents were stored at −20°C. Stock solutions were diluted to an appropriate concentration in culture medium before use. The siRNAs were purchased from Bioneer (AccuTarget™ negative-control siRNA, SN-1002; AccuTarget™ Human genome-wide predesigned siRNA, no. 111838 (RIPK1), 1096542 (RIPK3), and 1129536 (MLKL), Daejeon, South Korea).

### MTT assay

The cells were plated in 96-well plates at a density of 2 × 10^4^ cells per well. After incubating overnight, the cells were treated with HPA3, HPA3P, HPA3P2, oxaliplatin, and 5-FU at various concentrations. After the cells had incubated for 6 h or 24 h, the culture medium was replaced with fresh medium. Twenty microlitres of a 5 mg/ml stock solution of 3-(4,5-dimetylthiazol-2-yl)-2,5-diphenyltetrazolium bromide (Sigma Aldrich) was added to each well, and the cells were incubated for 1 h at 37°C in a humidified atmosphere of 5% CO_2_. After removal of the supernatant, 100 μl of DMSO was added to each well. The plates were subsequently incubated on a horizontal shaker for 20 min at room temperature, and the optical density was measured at an absorbance of 570 nm using a microplate reader (SPECTRA MAX PLUS, Molecular Devices, CA, USA).

### Soft agar assay

Anchorage-independent cell growth in soft agar was examined using colony formation assay in 12-well plates. A base layer containing 0.75% agarose in culture medium was added to each well, after which the plates were incubated at room temperature until the agar solidified. Then, 1 × 10^5^ cells were suspended in culture medium containing 0.4% agarose with HPA3P and immediately added atop the base layer. After the cells had incubated for 10 days, their colonies were fixed by methanol/acetone (10%:10%) and stained with 0.005% crystal violet. The colonies were subsequently photographed under a Nikon SMZ800 stereomicroscope (Nikon, Tokyo, Japan) using a digital camera and counted using ImageJ software.

### Flow cytometry

Cell death was assessed by annexin V and propidium iodide (PI) staining using an FITC Annexin V/Dead Cell Apoptosis Kit (Molecular Probes, OR, USA), according to the manufacturer's protocol. Briefly, the cells were plated in a 6-well plate at a density of 8 × 10^5^ cells per well and treated with HPA3P for 6 or 24 h. The cells were then trypsinized, washed with PBS, and resuspended in annexin V binding buffer. Specifically, the cells were treated with 5 μl of FITC-conjugated annexin V and 1 μl of 100 μg/ml PI solution and then incubated at room temperature for 15 min. The cells were subsequently analysed using a CytoFLEX flow cytometer (Beckman Coulter Inc., CA, USA).

### Western blot analysis

The cells were plated in 6-well plates at a density of 1 × 10^6^ cells per well. After incubating overnight, the cells were treated with HPA3P at the indicated concentration. After incubating for 6 h or 24 h, the cells were scraped, and the proteins were quantified by the Bradford method. The proteins were resolved on SDS-PAGE and transferred to PVDF membranes (Millipore, Bedford, USA). The blots were blocked with TBST containing 5% skim milk for 1 h, after which the membranes were incubated overnight at 4°C with primary antibodies against cleaved caspase-3 (#9664), PARP (#9542, Cell Signaling Technology, MA, USA), HMGB1 (ab18256, Abcam, Cambridge, UK), and GAPDH (SC-25778, Santa Cruz, CA, USA). The blots were then washed with TBST buffer and incubated with a secondary horseradish peroxidase-conjugated antibody (Santa Cruz, CA, USA). The reaction was detected using a Clarity™ Western ECL Substrate (BioRad, CA, USA).

### Fluorescence microscopy

The cells were seeded in a cell culture slide at a density of 4 × 10^4^ cells per well and treated with HPA3P for 30 min before being washed with PBS and incubated with Hoechst 33342 (Molecular Probes, OR, USA) and PI for 15 min at room temperature. Then cells were then washed with PBS and fixed 4% paraformaldehyde for 10 min. To investigate the subcellular localization of rhodamine-labelled HPA3P in live cells, we incubated the cells with rhodamine-labelled HPA3P for 5 min before washing them and incubating them with Hoechst 33342. The cells were subsequently incubated with wheat germ agglutinin conjugated with Alexa Fluor^®^ 594 (Molecular Probes, OR, USA) and Hoechst 33342 for 10 min, after which they were washed with PBS and treated HPA3P for 5 min. The slides were photographed with a fluorescence microscope (Olympus IX71, Tokyo, Japan).

### Measurement of peptide penetration

HCT116 p53^+/+^ cells were plated in 96-well plates at a density of 3 × 10^4^ cells in each well. After incubating overnight, the cells were treated with 30 μM rhodamine-labelled HPA3P. The plate was placed into an IncuCyte ZOOM™ system (Essen BioSciences, Ann Arbor, MI, USA) within a cell culture incubator. Four different areas per well in three wells were observed every 2 min for approximately 20 min (magnification 10X). Penetration and the images were analysed using IncuCyte ZOOM 2016A software.

### Transfection

HCT116 p53^+/+^ cells were plated in 96- and 6-well plates at a density of 2 × 10^4^ and 1.4 × 10^6^ cells per well, respectively. After incubating overnight, the cells were transfected with negative-control, RIPK1, RIPK3, and MLKL siRNA using Lipofectamine 2000 transfection reagent (Invitrogen Life Technologies, Carlsbad, CA, USA), according to the manufacturer's instructions. Briefly, the negative-control, RIPK1, RIPK3, and MLKL siRNAs and Lipofectamine 2000 were diluted in 50 and 250 μl Opti-MEM I, respectively, and incubated for 5 min at room temperature. Then, the diluted siRNAs and Lipofectamine 2000 were combined into a single solution. The solution was allowed to incubate for 20 min at room temperature, after which the resultant siRNA/Lipofectamine 2000 complexes were added to each well of the above plates. The cells were subsequently incubated at 37°C in a humidified atmosphere of 5% CO_2_. The concentrations of the siRNAs were optimized to 15 and 200 pmol per well in the 96- and 6-well plates, respectively. After 24 h of transfection, the cells were treated with HPA3P at the indicated concentrations for 6 h. Cell viability was analysed by MTT assay, and protein expression was analysed by western blotting with primary antibodies against the following proteins: RIPK1 (SC-133102, Santa Cruz, CA, USA), RIPK3 (SC-374639, Santa Cruz, CA, USA), and MLKL (H00197259-M02, Abnova, Taipei, Taiwan).

### Mouse tumour model

Male BALB/c nude mice aged 5 weeks were purchased from Orient Bio Inc. (Gyeonggi, South Korea). To establish the subcutaneous tumour model, we subcutaneously injected 1 × 10^7^ HCT116 p53^+/+^ cells into the right flanks of the mice. After 10 days, the mice were treated with HPA3P at a dose of 25 mg/kg or PBS via tail vein injection once daily for two days and then once four days thereafter. Tumour size was measured daily using a Vernier calliper, and tumour volumes were calculated using the formula: V (mm^3^) = L × W^2^/2, where L is the length of the tumour, and W is the width of the tumour. All animal experiments were performed according to the guidelines of the Chosun University Institutional Animal Care and Use Committee, and the protocol for the animal experiments was approved by the Animal Ethics Committee of Chosun University (Approval number: CIACUC2016-S0028).

### Statistical analysis

All data are presented as the mean ± SE of the mean or SD. Statistical analyses were performed using paired *t*-test or ANOVA followed by Tukey's post-hoc analysis. Between-group differences with a *p* < 0.05 were considered significant. All statistical analyses were performed using IBM SPSS Statistics version 20. Plotting graph was generated using GraphPad prism 6 (GraphPad Software Inc., CA, USA).

## SUPPLEMENTARY MATERIALS FIGURES AND TABLE






