# Supplementary material for: Antitumor activity of HPA3P through RIPK3-dependent regulated necrotic cell death in colon cancer
Source: Oncotarget. 2018 Jan 9;9(8):7902–17. doi: 10.18632/oncotarget.24083 (PMC5814268; doi:10.18632/oncotarget.24083)
Supplement: Supplementary file 1 [file oncotarget-09-7902-s001.pdf]

Antitumor activity of HPA3P through RIPK3-dependent regulated necrotic cell death in colon cancer

SUPPLEMENTARY MATERIALS

Confocal laser-scanning microscopy

The cells were seeded in an 8-well chamber slide at a density of 2 × 10^4 cells per well. After incubating overnight, the cells were washed with PBS. Then, the culture medium was replaced with fresh cell culture medium containing 10 μM rhodamine-labelled HPA3P for 15 min. The medium was subsequently removed, and the cells were washed three times with PBS. The cells were then fixed with 4% paraformaldehyde for 30 min at room temperature and washed three times with PBS before being treated with 0.1% Triton X-100 for 20 min at room temperature and washed with PBS. The nuclei were stained with ProLong® Gold Antifade Reagent with DAPI solution. Finally, the cells were washed three times with PBS, and the slides were mounted with coverslips. Rhodamine-labelled HPA3P localization was performed using an inverted LSM510 laser-scanning microscope (Carl Zeiss, Göttingen, Germany). The resulting images were recorded digitally in a 512 × 512 pixel format.

Quantitative real-time PCR (qRT-PCR)

Total RNA was extracted from cells using TRIzol reagent (Invitrogen) according to the manufacturer's protocol. The extracted RNA was quantified by a NanoDrop 2000 spectrophotometer (Thermo Scientific, Waltham, MA, USA). First-strand cDNA was synthesized from RNA for 60 minutes at 50° C using TOPscriptTM RT DryMIX (Enzynomics, Daejeon, Korea). Quantitative PCR was performed using TOPrealTM qPCR 2X PreMIX (SYBR Green with low ROX) (Enzynomics, Daejeon, Korea) on an ABI 7500 Real Time PCR System (Applied Biosystems, Carlsbad, CA, USA). All reactions were performed in triplicate for each sample. Transcript levels were calculated using the comparative Ct method and normalized to glyceraldehyde-3-phosphate dehydrogenase. The following primers were used: RIPK3, 5′-TGCTGGAAGAGAAGTTGAGTTG-3′ and 5′-CTGTTGCACACTGCTTCGTACAC-3′ [1]; GAPDH, 5′-GAAGGTGAAGGTCGGAGTCA-3′ and 5′-TTGAGGTCAATGAAGGGGTC-3′ [2].

REFERENCES

1. Harris KG, Morosky SA, Drummond CG, Patel M, Kim C, Stolz DB, Bergelson JM, Cherry S, Coyne CB. RIP3 Regulates Autophagy and Promotes Coxsackievirus B3 Infection of Intestinal Epithelial Cells. Cell Host Microbe. 2015; 18:221–32. https://doi.org/10.1016/j.chom.2015.07.007.
2. Roelofs HM, Te Morsche RH, van Heumen BW, Nagengast FM, Peters WH. Over-expression of COX-2 mRNA in colorectal cancer. BMC Gastroenterol. 2014; 14:1. https://doi.org/10.1186/1471-230x-14-1.

Supplementary Table 1: 95% confidence interval (CI) of penetrated Rho-HPA3P in panel B of Figure 4

Time (min)	Mean ± SE	95% CI	*P* value
0	88 ± 18.06	10.5–166	$p < 0.05$
2	170 ± 15.35	104.1–236.3	$p < 0.01$
4	368.33 ± 7.95	334.1–402.5	$p < 0.001$
6	575.21 ± 8.4	539–611.3	$p < 0.001$
8	725.5 ± 10.94	678.4–772.5	$p < 0.001$
10	833.21 ± 7.07	802.7–863.63	$p < 0.001$
12	933.57 ± 15.52	895–972.1	$p < 0.001$
14	1023.45 ± 10.92	976.4–1070.4	$p < 0.001$
16	1089.7 ± 3.17	1076–1103.3	$p < 0.001$
18	1165.31 ± 7.17	1134.4–1196.1	$p < 0.001$
20	1232.72 ± 4.4	1213.7–1251.6	$p < 0.001$
22	1283.53 ± 4.74	1263.1–1303.9	$p < 0.001$

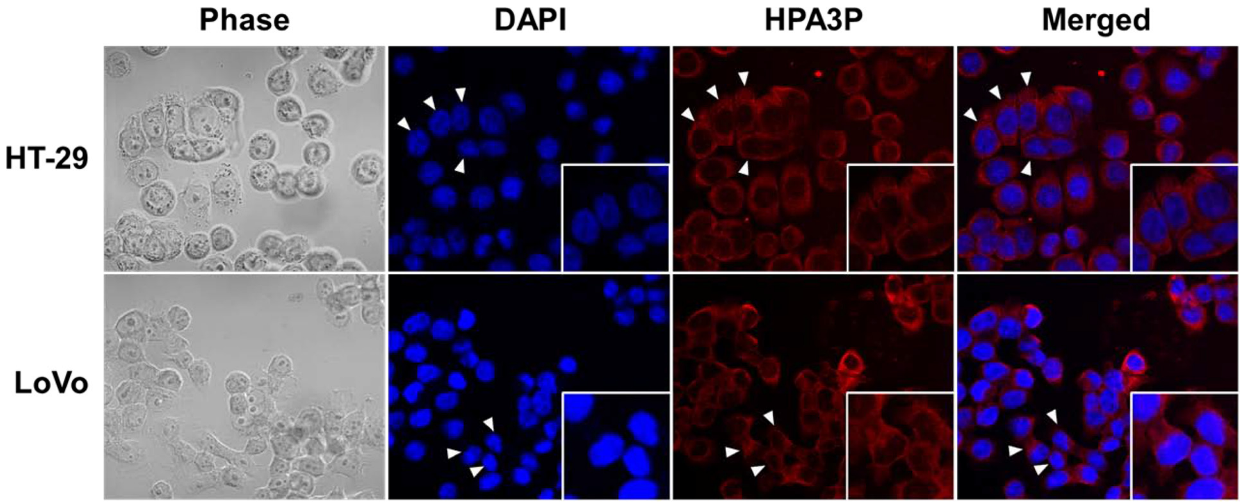

Supplementary Figure 1: HPA3P is located in the cytoplasm in HT-29 cells. Cells were treated with 10 µM rhodamine-labelled HPA3P for 15 min. Cell lines were washed, and then stained with DAPI. Images were photographed under an inverted LSM510 laser-scanning microscope (scale bar : 20 um, white box-scale bar : 5 um).

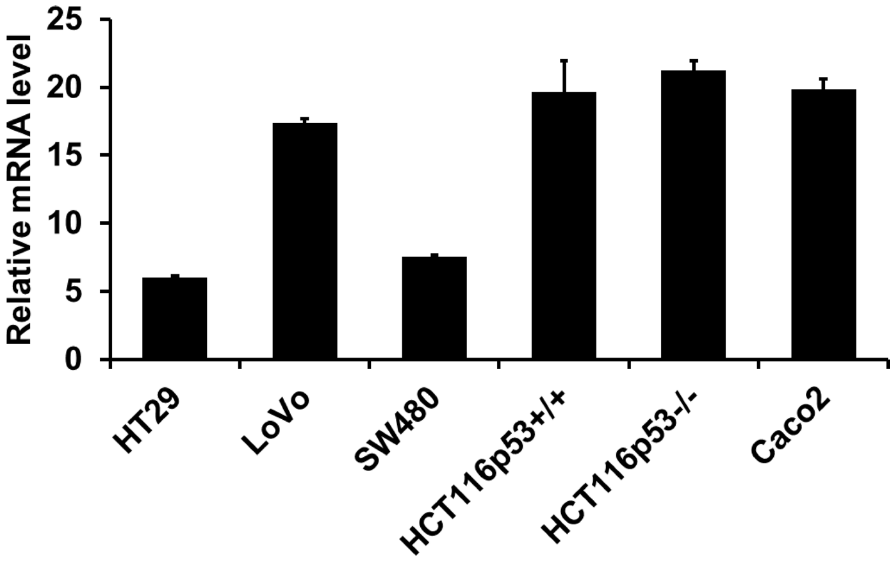

Supplementary Figure 2: mRNA level of RIPK3 in colon cancer cells. Total RNA was isolated from colon cancer cells. The expression of RIPK3 mRNA was assessed by real-time PCR. Data are shown as the mean ± SD.

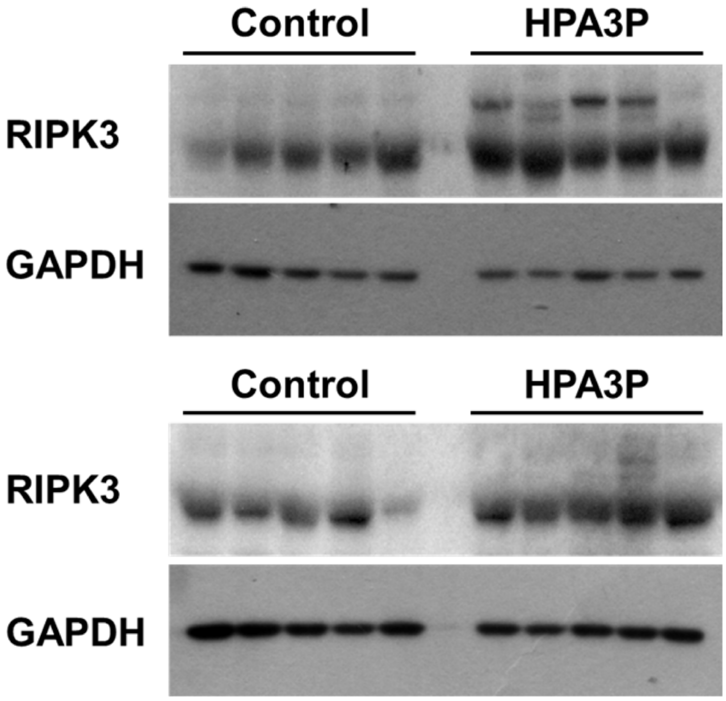

Supplementary Figure 3: The expression of RIPK3 in tumour tissues of mouse tumor models. HCT116 p53+/+ cells were injected into the right flanks of the nude mice. Ten days after implantation, the mice were treated with 25 mg/kg HPA3P and PBS by intravenous injection. On day 20 after tumour resection, RIPK3 expression in tumour tissue extract was analysed by western blotting and protein samples of each group were randomly loaded.

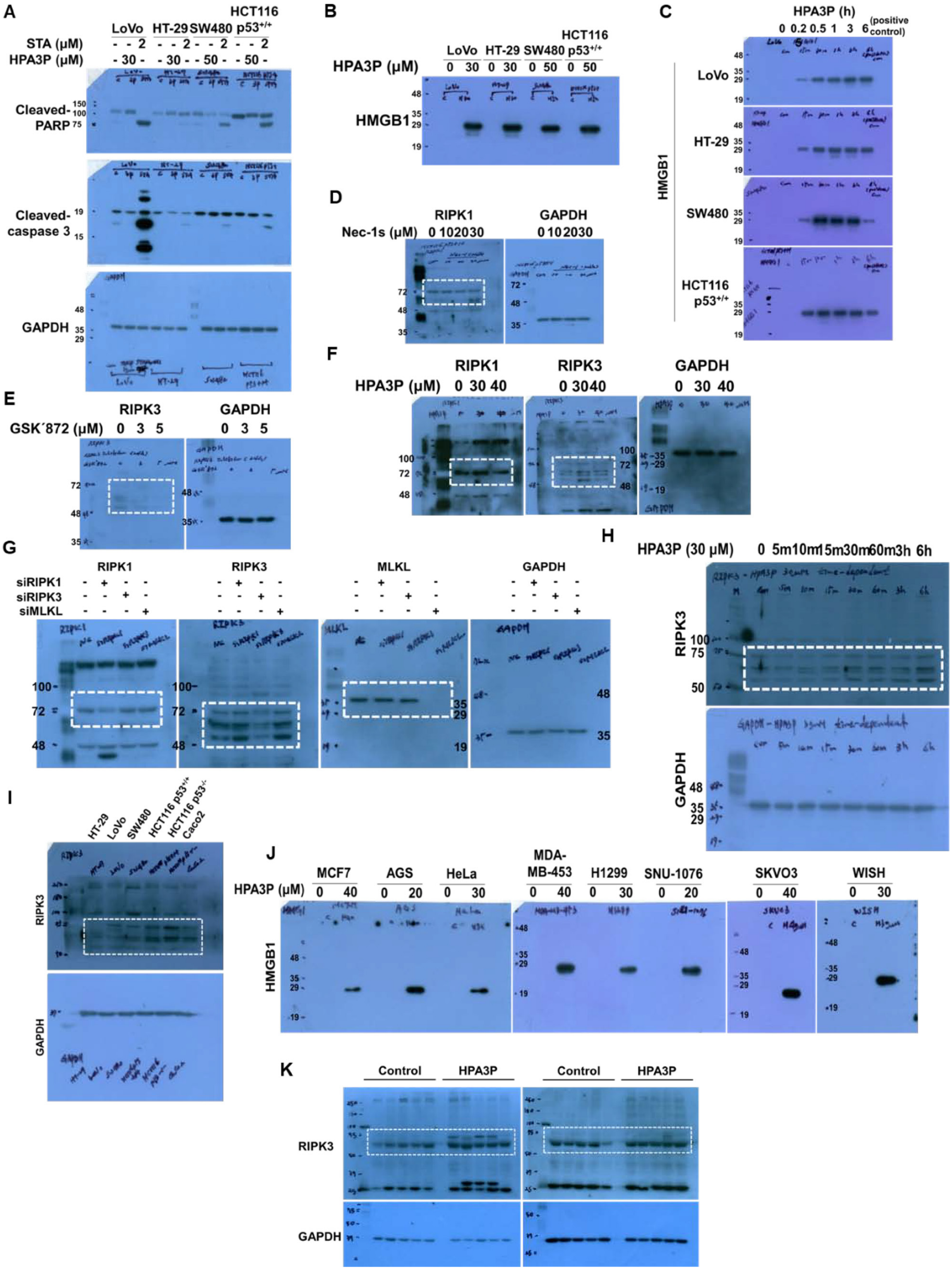

Supplementary Figure 4: Uncropped western blotting images.

Supplementary Video 1: Morphological changes by HPA3P in colon cancer cells. The indicated cell lines were treated with HPA3P (LoVo and HT-29, 30 μM/ml; SW480 and HCT116 $p53^{+/+}$, 50 μM/ml) for approximately 20 min. Immediately after treatment, a video was recorded using an Olympus IX71 fluorescence microscope (magnification PlanC N 40X). See Supplementary_Video_1

Supplementary Video 2: Penetration of HPA3P into HCT116 $p53^{+/+}$ cells. HCT116 $p53^{+/+}$ cells were treated with 30 μM/ml rhodamine-labelled HPA3P, and then fluorescence was measured for 20 min using an IncuCyte ZOOM imaging system. See Supplementary_Video_2
