# Supplementary material for: Antitumor activity of HPA3P through RIPK3-dependent regulated necrotic cell death in colon cancer
Source: Oncotarget. 2018 Jan 9;9(8):7902–17. doi: 10.18632/oncotarget.24083 (PMC5814268; doi:10.18632/oncotarget.24083)
Supplement: Supplementary file 2 [file oncotarget-09-7902-s002.pptx]

## Slide 1
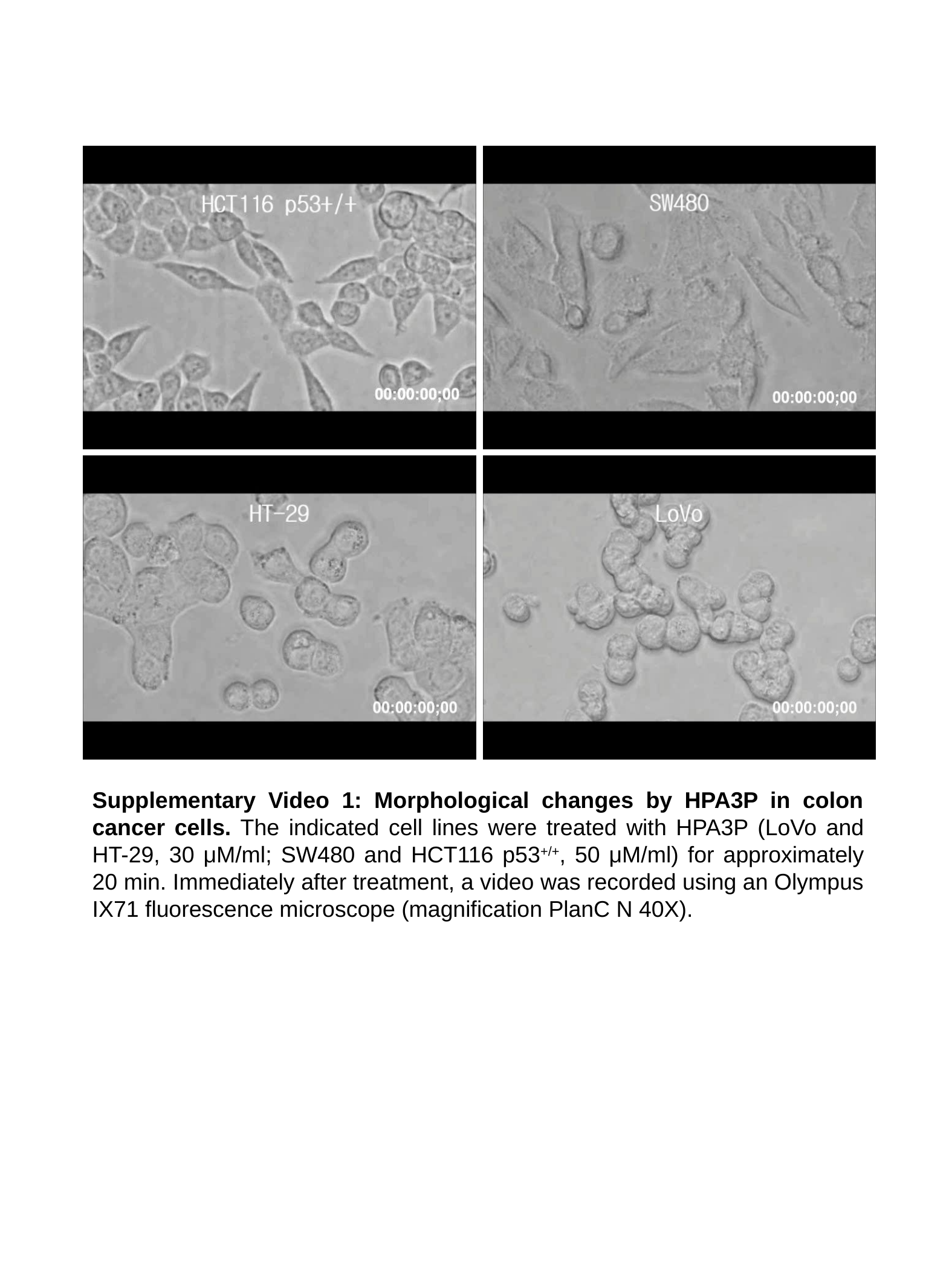

Supplementary Video 1: Morphological changes by HPA3P in colon cancer cells. The indicated cell lines were treated with HPA3P (LoVo and HT-29, 30 μM/ml; SW480 and HCT116 p53+/+, 50 μM/ml) for approximately 20 min. Immediately after treatment, a video was recorded using an Olympus IX71 fluorescence microscope (magnification PlanC N 40X).
