# Supplementary material for: Antitumor activity of HPA3P through RIPK3-dependent regulated necrotic cell death in colon cancer
Source: Oncotarget. 2018 Jan 9;9(8):7902–17. doi: 10.18632/oncotarget.24083 (PMC5814268; doi:10.18632/oncotarget.24083)
Supplement: Supplementary file 3 [file oncotarget-09-7902-s003.pptx]

## Slide 1
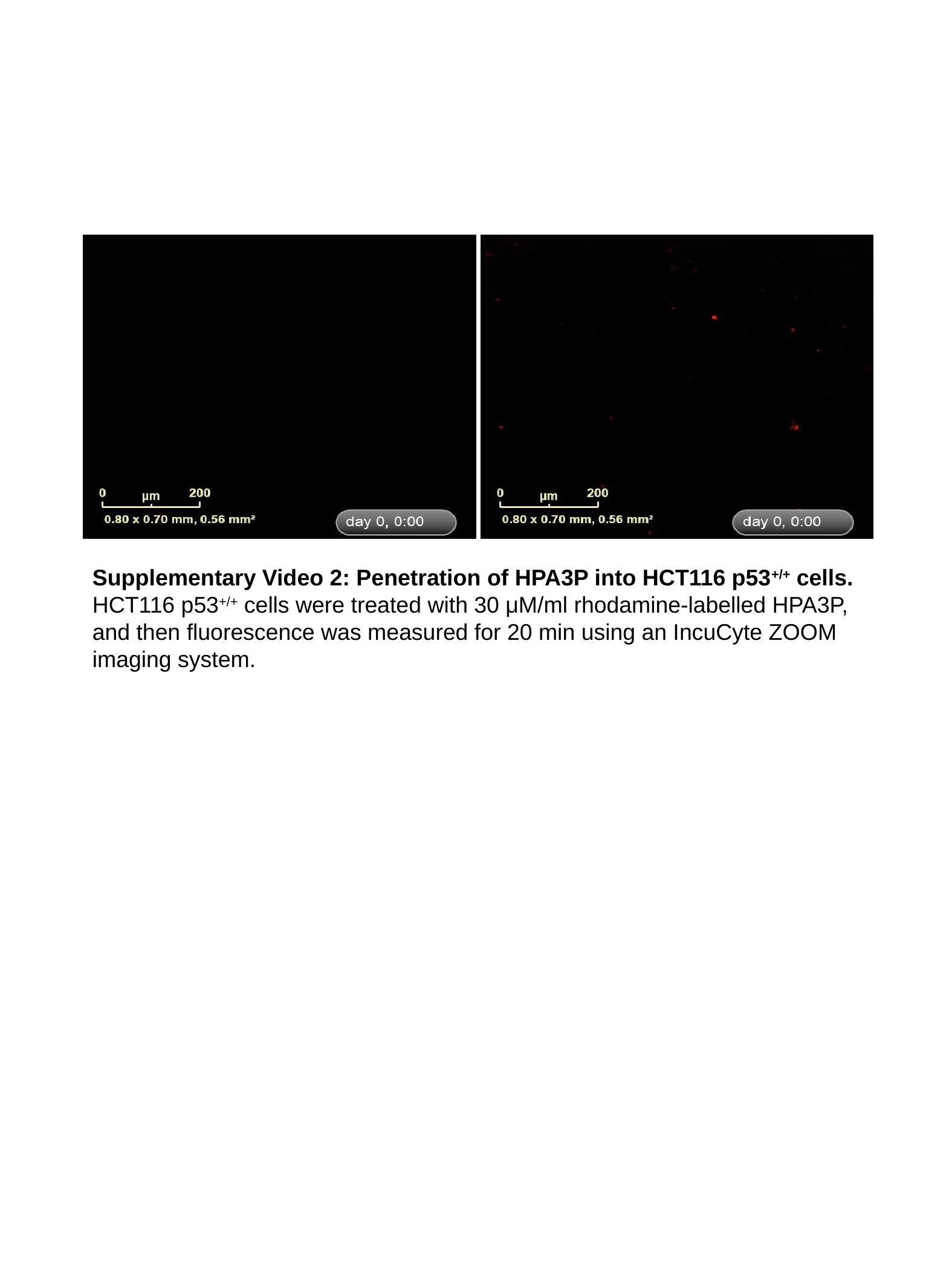

Supplementary Video 2: Penetration of HPA3P into HCT116 p53+/+ cells. HCT116 p53+/+ cells were treated with 30 μM/ml rhodamine-labelled HPA3P, and then fluorescence was measured for 20 min using an IncuCyte ZOOM imaging system.
